# Exploration of risk factors for ceftriaxone resistance in invasive non-typhoidal *Salmonella* infections in western Kenya

**DOI:** 10.1371/journal.pone.0229581

**Published:** 2020-03-03

**Authors:** Ulzii-Orshikh Luvsansharav, James Wakhungu, Julian Grass, Martina Oneko, Von Nguyen, Godfrey Bigogo, Eric Ogola, Allan Audi, Dickens Onyango, Mary J. Hamel, Joel M. Montgomery, Patricia I. Fields, Barbara E. Mahon

**Affiliations:** 1 Epidemic Intelligence Service, Epidemiology Workforce Branch, Division of Scientific Education and Professional Development, Centers for Disease Control and Prevention, Atlanta, Georgia, United States of America; 2 Enteric Diseases Epidemiology Branch, Division of Foodborne, Waterborne and Environmental Diseases, National Center for Emerging Zoonotic Infectious Diseases, Centers for Disease Control and Prevention, Atlanta, Georgia, United States of America; 3 Field Epidemiology and Laboratory Training Program, Ministry of Health, Nairobi, Kenya; 4 Centre for Global Health Research, Kenya Medical Research Institute, Nairobi, Kenya; 5 Ministry of Health, Nairobi, Kenya; 6 Malaria Branch, Division of Parasitic Diseases and Malaria, Centers for Disease Control and Prevention, Atlanta, Georgia, United States of America; 7 Division of Global Health Protection, Centers for Disease Control and Prevention, Nairobi, Kenya; 8 Enteric Diseases Laboratory Branch, Division of Foodborne, Waterborne and Environmental Diseases, National Center for Emerging Zoonotic Infectious Diseases, Centers for Disease Control and Prevention, Atlanta, Georgia, United States of America; University of Georgia, UNITED STATES

## Abstract

Multidrug-resistant non-typhoidal *Salmonella* (NTS) infection has emerged as a prominent cause of invasive infections in Africa. We investigated the prevalence of ceftriaxone-resistant invasive NTS infections, conducted exploratory analysis of risk factors for resistance, and described antimicrobial use in western Kenya. We conducted a secondary analysis of existing laboratory, epidemiology, and clinical data from three independent projects, a malaria vaccine trial, a central nervous system (CNS) study, and the International Emerging Infections Program morbidity surveillance (surveillance program) during 2009–2014. We calculated odds ratios (OR) with 95% confidence intervals (CI) for ceftriaxone-resistant NTS infections compared with ceftriaxone-susceptible infections. We surveyed hospitals, pharmacies, and animal drug retailers about the availability and use of antimicrobials. In total, 286 invasive NTS infections were identified in the three projects; 43 NTS isolates were ceftriaxone-resistant. The absolute prevalence of ceftriaxone resistance varied among these methodologically diverse projects, with 18% (16/90) of isolates resistant to ceftriaxone in the vaccine trial, 89% (16/18) in the CNS study, and 6% (11/178) in the surveillance program. Invasive ceftriaxone-resistant infections increased over time. Most ceftriaxone-resistant isolates were co-resistant to multiple other antimicrobials. Having an HIV-positive mother (OR = 3.7; CI = 1.2–11.4) and taking trimethoprim-sulfamethoxazole for the current illness (OR = 9.6, CI = 1.2–78.9) were significantly associated with acquiring ceftriaxone-resistant invasive NTS infection. Ceftriaxone and other antibiotics were widely prescribed; multiple issues related to prescription practices and misuse were identified. In summary, ceftriaxone-resistant invasive NTS infection is increasing and limiting treatment options for serious infections. Efforts are ongoing to address the urgent need for improved microbiologic diagnostic capacity and an antimicrobial surveillance system in Kenya.

## Introduction

Non-typhoidal *Salmonella* (NTS) infection has emerged as a prominent cause of bloodstream infection in Africa with an associated case fatality of 20–25% [1, 2]. NTS is among the most common pathogens causing bacteremia in Africa [3, 4]. HIV-positive adults and young children carry most of the burden [3–5]. An extremely high burden of invasive NTS infections has been observed in rural western Kenya, with the highest adjusted annual incidence of 3,914.3 per 100,000 person-years of observation (2009–2014) among children younger than 5 years of age [6]. Additionally, multidrug-resistant NTS strains have emerged in Africa, which can lead to treatment failure and complicate patient management [7]. Recent emergence of resistance to extended-spectrum cephalosporins, such as ceftriaxone, is especially concerning because these drugs are important for treating invasive NTS infection in children, for whom fluoroquinolones are usually avoided [8]. Ceftriaxone resistance had not been documented in Kenya until recent years. However, investigators in Siaya county, western Kenya observed the emergence in 2009 and increase through 2013 of ceftriaxone-resistant invasive NTS infections in children participating in a malaria vaccine trial in whom blood culture was routinely performed to evaluate febrile illnesses [9].

In addition to the malaria vaccine trial, hereafter referred to as “the vaccine trial,” two other infectious disease projects conducted in the same county and period performed blood cultures for participants with febrile illnesses. These projects were 1) a study of central nervous system infections, hereafter called “the CNS study,” and 2) an ongoing morbidity surveillance of the International Emerging Infections Program, hereafter called “the surveillance program.” Using these data, we investigated the trajectory of prevalence of ceftriaxone-resistant invasive NTS infections and conducted exploratory analysis of potential risk factors for resistance. We also collected primary data about the use of antimicrobial agents that could have contributed to the selection of resistant NTS in humans and animals in western Kenya.

## Materials and methods

### Background on the three projects that contributed data to this analysis

Details of the vaccine trial, the CNS study, and the surveillance program have been described previously [9–11]; for background, we briefly summarize these three projects. First, the vaccine trial enrolled 1,696 children aged 6 weeks to 17 months from 2009 through 2013, and participants were followed for up to 53 months. Blood culture was performed for participants who were hospitalized or were evaluated for prolonged fever at outpatient clinics [9]. Second, the CNS study enrolled 320 participants aged ≥6 weeks who were hospitalized and met criteria for evaluation for acute central nervous system infection from November 2011 through December 2013 [10]. Blood and cerebrospinal fluid (CSF) culture were performed for all participants. Third, the surveillance program, which covers a population of about 25,000 persons, has conducted population-based infectious disease surveillance since 2005. Blood cultures are performed for outpatients meeting the case definitions for severe acute respiratory illness and acute febrile illness and for all inpatients admitted for conditions unrelated to injury or obstetric causes [11]. We analyzed surveillance program data from January 2009 through April 2014. The protocols for all three projects included routine culture of participant blood, and for all three projects, isolates were identified as NTS and serogrouped at the Kenya Medical Research Institute (KEMRI) laboratory in Kisumu using standard techniques [6, 9]. For all three projects, KEMRI assessed antimicrobial susceptibility using Kirby-Bauer disk diffusion methods and Clinical and Laboratory Standard Institute (CLSI) guidelines for determining resistance [12]. The detection of ceftriaxone-resistant serogroup O4 (formerly serogroup B) NTS isolates triggered the current investigation, and we conducted a secondary analysis of existing laboratory, epidemiology, and clinical data from the three projects described above.

### Secondary analysis of prevalence and risk factors for ceftriaxone resistance in invasive NTS infections

We conducted a secondary analysis of existing data on cases of laboratory-confirmed invasive NTS infections identified through the vaccine trial, the CNS study, and the surveillance program, including all cases in which a blood culture yielded NTS. If NTS was isolated more than once from a person, we included the first isolate, except in cases in which ceftriaxone-susceptible and ceftriaxone-resistant isolates were both identified, when we included the first ceftriaxone-resistant isolate. Using existing epidemiologic, clinical, and antimicrobial susceptibility testing (AST) data from the three projects, ceftriaxone-resistant NTS infections were compared to ceftriaxone-susceptible infections; for some variables, information was available from only one or two of the projects; the data available from each project is detailed in Table 1. We conducted univariate analysis to calculate odds ratios (OR) with 95% confidence intervals (CI) for ceftriaxone-resistant infections, as compared with ceftriaxone-susceptible infections. We compared categorical data using the χ^2^ test or Fisher’s exact test, as appropriate, and continuous data using the Mann-Whitney U-test. Statistical analyses were conducted using SAS 9.3 (SAS Institute, Cary, NC).

**Table 1:** Variables available for risk factor analysis for malaria vaccine trial (vaccine trial), central nervous system infection study (CNS study), and International Emerging Infections Program (surveillance program) participants during 2009–2014, Siaya county, Kenya.

Variables	Study		
	Vaccine trial	CNS study	Surveillance program
**Demographics**			
Age	√^a^	√	√
Gender	√	√	√
**Host and maternal factors**			
HIV status^b^	√		
HIV status of mother	√		
Malaria within 2 weeks before invasive NTS infection diagnosis^b^	√		
Malaria at the time of invasive NTS infection diagnosis	√	√	√
**Exposure to antimicrobials before visiting a clinic/hospital**			
Took antibiotics before arriving to hospital^b^		√	
Taken a medication for this illness^b^			√
Sulfadoxine/pyrimethamine^b^			√
Trimethoprim-sulfamethoxazole^b^			√
Penicillin^b^			√
Other antimicrobials^b^			√
**Clinical history and symptoms**			
Fever with this illness^b^		√	√
Temperature in Celsius	√	√	√
Stiff neck^b^		√	
Diarrhea	√	√	√
Vomiting^b^		√	√
**Outcome**			
Admitted to hospital	√	√	√
Duration of hospitalization^b^			√
Hospital admission outcome	√	√	√

CNS, central nervous system.

^**a**^ √-data available for analysis.

^b^blank-data not available for the current analysis.

### Surveys regarding availability and use of antimicrobial agents

We conducted surveys (S1, S2 and S3 Appendices) at 3 hospitals (including 7 healthcare worker respondents), 23 pharmacies (including 23 pharmacy staff respondents), and 15 agrovets (shops that supply drugs, feeds, and other products for livestock; including 15 staff respondents). We included all registered facilities in Lwak and Siaya towns in Siaya county. The surveys covered questions about the availability and administration of antimicrobials, how patients or their families purchase and use antimicrobials, common selling practices, the type of livestock that were commonly administered antimicrobials, and the presence of antimicrobials in animal feeds and supplements that were available for sale on the day of the survey. Participation was voluntary and anonymous, and we received verbal permission from all the survey participants.

### Further characterization of selected ceftriaxone-resistant NTS isolates

A convenience sample of 31 serogroup O4 NTS isolates (12 from the vaccine trial, 13 from the CNS study, and 6 from the surveillance program) was serotyped and tested for antimicrobial susceptibility using the broth microdilution method per CLSI guidelines in the Enteric Diseases Laboratory Branch (EDLB) at CDC [12, 13]. Whole genome sequencing was performed on 4 these isolates from the CNS study and the surveillance program using methods as described by Tagg K.A *et*.*al*. [14].

### Institutional approvals

The Kenyan Ministry of Health and CDC determined that the activities we conducted for this public health response to the emergence of ceftriaxone-resistant *Salmonella* infections did not meet the definition of research. Therefore, formal review by research ethics boards was not required for the activities we report.

## Results

### Prevalence and increase of ceftriaxone resistance in invasive NTS infections

**Fig 1:**
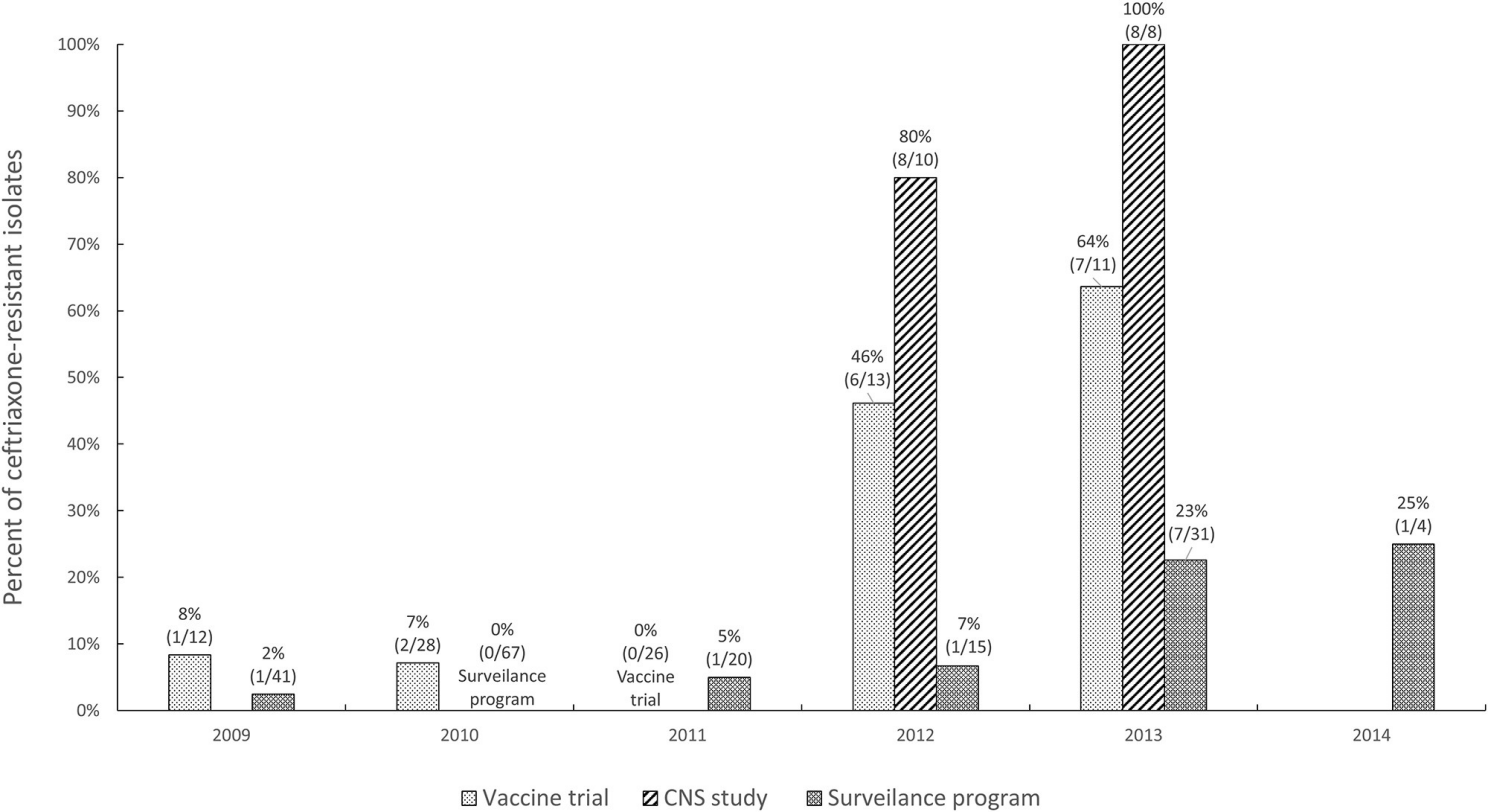
Percentage (number) of ceftriaxone-resistant isolates among invasive non-typhoidal *Salmonella* cases identified in malaria vaccine trial (vaccine trial; 2009–2013), central nervous system infection study (CNS study; 2012–2013), and International Emerging Infections Program (surveillance program; 2009–2014), Siaya county, Kenya.

In total, 286 invasive NTS infections were identified in the three projects; 43 NTS isolates were ceftriaxone-resistant (Table 2). The absolute prevalence of ceftriaxone resistance varied between these methodologically diverse projects, with 18% (16/90) of NTS isolates resistant to ceftriaxone in the vaccine trial, 89% (16/18) in the CNS study, and 6% (11/178) in the surveillance program (Table 2). The percentage of the NTS that was ceftriaxone-resistant increased over time in all three projects; from 8% (1/12) in 2009 to 64% (7/11) in 2013 for the vaccine trial, from 80% (8/10) in 2012 to 100% (8/8) in 2013 for the CNS study, and from 2% (1/41) in 2009 to 25% (1/4) in 2014 for the surveillance program (Fig 1). The NTS isolates that were resistant to ceftriaxone were also co-resistant to other antibiotics tested; all isolates were co-resistant to trimethoprim-sulfamethoxazole and sulfisoxazole (43/43 and 40/40, respectively), and almost all were co-resistant to ampicillin (98%, 42/43), gentamicin (95%, 41/43), tetracycline (95%, 38/40) and chloramphenicol (93%, 40/43) (Table 3).

**Table 2:** Characteristics of patients with invasive non-typhoidal *Salmonella* infections among malaria vaccine trial (vaccine trial), central nervous system infection study (CNS study), and International Emerging Infections Program (surveillance program) participants during 2009–2014, Siaya county, Kenya.

Characteristics	Study	Ceftriaxone-resistant infection n/N (%)	Ceftriaxone-susceptible infection n/N (%)	Odds ratio^a^ (95% confidence interval)
**Number of invasive NTS infection cases**	Vaccine trial	16/90 (18)	74/90 (82)	
	CNS study	16/18 (89)	2/18 (11)	
	Surveillance program	11/178 (6)	167/178 (94)	
**Demographics**				
Age in months, median (range)	Vaccine trial^b^	35 (4–53)	15 (2–47)	
	CNS study	16 (3–62)	15 (2–28)	
	Surveillance program	42 (18–437)	50 (4–918)	
Male gender	Vaccine trial	6/16 (38)	35/74 (47)	0.7(0.2–2.0)
	CNS study	10/16 (63)	0/2 (0)	8.1(0.3–196.2)
	Surveillance program	6/11 (55)	78/167 (47)	1.4(0.4–4.7)
**Host and maternal factors**				
HIV positive^c^	Vaccine trial	2/16 (13)	7/70 (10)	1.3(0.2–6.9)
Has an HIV-positive mother	Vaccine trial	9/16 (56)	19/74 (26)	3.7(1.2–11.4)
Had malaria within 2 weeks before invasive NTS infection diagnosis	Vaccine trial	10/16 (63)	46/74 (62)	1.0(0.3–3.1)
Had malaria at the time of invasive NTS infection diagnosis^c^	Vaccine trial	8/16 (50)	37/74 (50)	1.0(0.3–3.0)
	Surveillance program	3/5 (60)	14/49 (29)	3.8(0.6–24.9)
**Exposure to antimicrobials before visiting a clinic/hospital**				
Took antibiotics before arriving to hospital^c^	CNS study	5/9 (56)	1/1 (100)	0.4(0.0–12.6)
Taken a medication for this illness	Surveillance program	5/11 (45)	85/167 (51)	0.8(0.2–2.7)
Sulfadoxine/pyrimethamine^c^	Surveillance program	0/4 (0)	2/75 (3)	3.3(0.1–78.7)
Trimethoprim-sulfamethoxazole^c^	Surveillance program	2/4 (50)	7/74(9)	9.6(1.2–78.9)
Penicillin^c^	Surveillance program	0/4 (0)	8/76 (11)	0.9(0.0–18.1)
Other antimicrobials^c^	Surveillance program	0/4 (0)	3/74 (4)	2.3(0.1–51.0)
**Clinical history and symptoms**				
Fever^c^	CNS study	14/16 (88)	2/2 (100)	1.2(0.0–32.1)
	Surveillance program	11/11 (100)	157/166 (95)	1.4(0.1–25.4)
Temperature in Celsius, median (range) ^c^	Vaccine trial	38 (36–41)	38 (35–41)	
	CNS study	38 (36–40)	39 (39–39)	
	Surveillance program	39 (37–40)	39 (35–41)	
Stiff neck^c^	CNS study	7/15 (47)	1/2 (50)	0.9(0.0–16.7)
Diarrhea^c^	Vaccine trial	8/15 (53)	34/73 (47)	1.3(0.4–4.0)
	CNS study	6/14 (43)	2/2 (100)	0.2(0.0–3.8)
	Surveillance program	0/11 (0)	44/166 (27)	0.1(0.0–2.1)
Vomiting^c^	Surveillance program	1/11 (9)	48/166 (29)	0.2(0.0–2.0)
**Outcome**				
Admitted to hospital^c^	Surveillance program	5/11 (45)	49/166 (30)	2.0(0.6–6.8)
Duration of hospitalization, days (median, range) ^c^	Surveillance program	3 (2–7)	3 (0–9)	
Discharged without sequelae^c^	Surveillance program	4/4 (100)	46/49 (94)	0.7(0.0–15.3)
Death	Vaccine trial	0/16 (0)	3/74 (4)	0.6(0.0–12.6)

Data are presented as No. (%) unless otherwise indicated.

CNS, central nervous system.

^a^Calculated by X^2^ test or Fisher’s exact test, as appropriate.

^b^The older age of patients with ceftriaxone-resistant NTS infection among the vaccine trial participants may have been related to the emergence of resistance relatively late (2013 and 2014) in the study when the participants of this longitudinal study in general have grown older.

^c^Missing data were excluded from the analyses.

**Table 3:** Antibiotic resistance patterns of the non-typhoidal *Salmonella* isolates from invasive infections identified among malaria vaccine trial (vaccine trial), central nervous system infection study (CNS study), and International Emerging Infections Program (surveillance program) participants during 2009–2014, Siaya county, Kenya.

CLSI Antimicrobial Class	Antimicrobial^a^	Vaccine trial		CNS study		Surveillance program	
		Ceftriaxone-resistant isolates	Ceftriaxone-susceptible isolates	Ceftriaxone-resistant isolates	Ceftriaxone-susceptible isolates	Ceftriaxone-resistant isolates	Ceftriaxone-susceptible isolates
		n/N (%)	n/N (%)	n/N (%)	n/N (%)	n/N (%)	n/N (%)
Aminoglycosides	Gentamicin	16/16 (100)	0/74 (0)	16/16 (100)	0/2 (0)	9/11 (82)	5/154 (3)
	Kanamycin	14/15 (93)	1/74 (1)	16/16 (100)	0/0	9/11 (82)	11/154 (7)
	Streptomycin	6/6 (100)	59/64 (92)	15/16 (94)	2/2 (100)	11/11 (100)	138/155 (89)
β-lactam/β-lactamase inhibitor combinations	Amoxicillin-clavulanic acid	16/16 (100)	34/66 (52)	16/16 (100)	1/2 (50)	6/10 (60)	62/146 (42)
Cephems	Cefotaxime	1/1 (100)	0/1 (0)	0/0	0/0	0/0	0/0
Folate pathway inhibitors	Sulfisoxazole	13/13 (100)	34/40 (85)	16/16 (100)	2/2 (100)	11/11 (100)	140/154 (91)
	Trimethoprim-sulfamethoxazole	16/16 (100)	67/74 (91)	16/16 (100)	2/2 (100)	11/11 (100)	137/156 (88)
Penicillins	Ampicillin	16/16 (100)	63/70 (90)	15/16 (94)	2/2 (100)	11/11 (100)	135/155 (87)
Phenicols	Chloramphenicol	16/16 (100)	57/74 (77)	14/16 (88)	2/2 (100)	10/11 (91)	123/156 (79)
Quinolones	Ciprofloxacin	1/16 (6)	0/73 (0)	3/16 (19)	0/2 (0)	1/11 (9)	0/155 (0)
	Nalidixic acid	1/16 (6)	6/73 (8)	2/16 (13)	0/2 (0)	3/11 (27)	26/155 (17)
Tetracyclines	Tetracycline	13/13 (100)	12/24 (50)	15/16 (94)	0/2 (0)	10/11 (91)	60/156 (38)

Data are presented as No. (%) unless otherwise indicated.

CLSI, Clinical and Laboratory Standards Institute; CNS, central nervous system.

^a^Not all isolates were tested for every antimicrobial listed.

### Risk factors for acquiring ceftriaxone-resistant invasive NTS infection

#### Demographic characteristics

There were no statistically significant differences in the median age of the CNS study and surveillance program participants with ceftriaxone-resistant isolates and ceftriaxone-susceptible isolates (16 months vs. 15 months, and 42 months vs. 50 months, respectively). Because of the longitudinal follow-up in the vaccine trial and the timing of the emergence of ceftriaxone resistance, the patients with ceftriaxone-resistant invasive NTS infections in the vaccine trial were older than patients with ceftriaxone-susceptible invasive NTS infections (median age 35 months vs. 15 months, respectively). The gender distribution of participants with ceftriaxone-resistant and ceftriaxone-susceptible NTS infection was similar in all programs. (Table 2).

#### Host and maternal factors

Having an HIV-positive mother was the only maternal factor significantly associated with ceftriaxone-resistant NTS infection, with significantly higher risk of resistant infection in the vaccine trial participants with HIV-positive mothers than in those with HIV-negative mothers (OR = 3.7; CI = 1.2–11.4). However, ceftriaxone-resistant or susceptible invasive NTS infection was not found to be associated with HIV status of the participants themselves (OR = 1.3; CI = 0.2–6.9). The proportion of participants with malaria in the two weeks before or at the time of invasive NTS infection was similar in ceftriaxone-resistant and -susceptible invasive NTS infection (OR = 1.0; CI = 0.3–3.1 and OR = 1.0; CI = 0.3–3.0, respectively) (Table 2).

#### Exposure to antibiotics

Administration of trimethoprim-sulfamethoxazole, but not other antimicrobials, for the current illness was significantly associated with ceftriaxone-resistant invasive NTS infection in the surveillance program participants (OR = 9.6, CI = 1.2–78.9) (Table 2). The overall percentage of the surveillance program patients who took any medication for the current illness before arriving to the hospital or clinic did not differ between ceftriaxone-resistant and -susceptible invasive NTS infection patients (OR = 0.8; CI = 0.2–2.7).

### Clinical presentation and outcomes

Although not statistically significant, presentation with diarrhea and vomiting was more common in patients with ceftriaxone-susceptible invasive NTS infections than ceftriaxone-resistant NTS infections. The percentage of surveillance program participants who were hospitalized and the duration of their hospital stay did not differ between patients with ceftriaxone-resistant and ceftriaxone-susceptible invasive NTS infections. No deaths were reported among the patients with ceftriaxone-resistant infection.

### Survey findings regarding antimicrobial availability and use

Kenyan informants reported that the third-generation cephalosporins first became available as early as 2005 in western Kenya. According to hospital respondents, the third-generation cephalosporins were “always” prescribed for children admitted with suspected meningitis (5 of 7 responders) and for those with suspected sepsis (3/7), and “often” prescribed for patients with acute febrile illnesses (2/7) (S1 Table). Interviews with clinicians revealed several issues related to prescription practices and adherence to prescribed dosages, including drug supply shortages at hospitals requiring inpatients to purchase drugs from outside pharmacies, antimicrobial purchase without a prescription, self-medication, and using antimicrobials in sub-therapeutic doses. In addition to ceftriaxone, clinicians commonly prescribed other antimicrobials, such as amoxicillin, trimethoprim-sulfamethoxazole and gentamicin, to which the ceftriaxone-resistant NTS was found to be co-resistant. Carbapenem antimicrobials were not available at any of the hospitals on the day of interview.

All of the 23 interviewed pharmacist dispensed antimicrobials, and 20 of them sold third-generation cephalosporins (S2 Table). Other beta-lactam antibiotics were available at 22 pharmacies, trimethoprim-sulfamethoxazole at 21, gentamicin at 19, and ciprofloxacin at 19 on the day of interview. Two of the 23 pharmacies had carbapenems available on the day of interview. Of the 23 pharmacies, respondents for 13 reported that they would sell a partial prescription to a customer. During the interviews, we observed that people could purchase antibiotics without a prescription at some pharmacies.

The practice of dispensing antimicrobials for veterinary use was similar to that for human use; antibiotic purchase without a prescription, treatment without veterinarian’s consult and subtherapeutic dosing were common. Agrovets also sold a wide range of antimicrobials for animal use to which ceftriaxone-resistant NTS in humans were co-resistant; all or most of the 15 interviewed agrovets sold sulfonamides (15), beta-lactams (14), tetracyclines (13) and macrolides (13) (S3 Table). In addition to antimicrobials, more than half of commonly sold brands of poultry nutritional supplements had tetracycline or oxytetracycline additives (15 of 27 observed supplements) that could contribute to co-selection of ceftriaxone-resistance.

### Further characterization of selected ceftriaxone-resistant NTS isolates

All 31 ceftriaxone-resistant serogroup O4 NTS isolates referred from the KEMRI laboratory to CDC for further characterization were serotype Typhimurium (S4 Table). Whole genome sequencing of 4 ceftriaxone-resistant NTS isolates from the CNS study and the surveillance program identified several plasmid types and multidrug resistance genes, including *bla*_CTX-M-15_ that confers resistance to broad spectrum cephalosporins and several folate pathway inhibitor genes (*dfrA1*, *dfrA14*, *sul1*, *sul2)* that confer resistance to trimethoprim-sulfamethoxazole. All four isolates were a single multilocus sequence type, ST313.

## Discussion

Ceftriaxone-resistant invasive NTS infections rapidly emerged during 2009–2014 in this area of western Kenya, where malaria and HIV are endemic (Fig 1). The true prevalence of multidrug-resistant NTS infections may be even larger than the prevalence these research and surveillance programs identified, because many infections are undetected due to lack of microbiologic diagnostic capacity in standard clinical settings and the absence of surveillance systems that track antimicrobial resistance in Kenya [15].

Treatment options for invasive ceftriaxone-resistant NTS infections are limited because most of the isolates were co-resistant to multiple antimicrobials, including ciprofloxacin (6% [1/16], 19% [3/16], and 1/11 [9%] of isolates identified in vaccine trial, CNS study and surveillance program, respectively) for some isolates. Similar findings of emerging multidrug resistance among invasive NTS infection isolates were reported from multiple African countries [7, 16]. Carbapenems and tigecycline are treatment alternatives for invasive NTS infections that are resistant to both first- and second-line drugs (amoxicillin, chloramphenicol, trimethoprim-sulfamethoxazole, extended-spectrum cephalosporins, and fluoroquinolones) [8]. Carbapenems were not available in the surveyed hospitals in western Kenya at the time of survey, and we did not inquire about tigecycline availability at the time of the study. Only the vaccine trial investigators had carbapenems available to treat their participants; the drug had been purchased and transported from a pharmacy in Nairobi. However, according to a later survey on antibiotic use, carbapenems became available in western Kenya [17]. Despite the limited treatment options, no deaths were reported among the patients with ceftriaxone-resistant NTS infection. Early detection and diagnosis, timely microbiological results, immediate medical care provided through the research and surveillance programs, and availability of appropriate antimicrobial therapy (vaccine trial), may have improved outcomes.

In our examination of risk factors, we found that having taken trimethoprim-sulfamethoxazole for the current illness or having an HIV-positive mother were each significantly associated with having ceftriaxone-resistant invasive NTS infection. All the ceftriaxone-resistant NTS isolates were co-resistant to trimethoprim-sulfamethoxazole, and the whole genome sequencing of the 4 ceftriaxone-resistant NTS isolates identified several plasmid types and multiple resistance genes that confer resistance to both drugs. A previous study reported that ceftriaxone-resistant NTS isolates in Nairobi harbored ceftriaxone resistance and multidrug resistance genes on a single plasmid [18]. Thus, it is possible that exposure to trimethoprim-sulfamethoxazole co-selected for ceftriaxone resistance. Furthermore, Kenyan national manual for management of HIV-related opportunistic infections and conditions recommends daily trimethoprim-sulfamethoxazole for all HIV-infected persons to prevent opportunistic infections [19]. Although we did not have information on mothers’ antibiotic use or their carriage of ceftriaxone-resistant organisms, HIV-positive mothers may have been on daily trimethoprim-sulfamethoxazole prevention and may have harbored and passed multidrug-resistant bacteria to their children. Household transmission and intrafamilial co-carriage of resistant *Enterobacteriaceae* have been documented in previous studies [20–22].

Our exploratory analysis of existing data did not identify other risk factors associated with ceftriaxone resistance; the small number of isolates in each study and the varying study designs limited our ability to assess associations. Also, these studies were conducted in a limited geographical area and the results may not be generalized to other areas. Furthermore, these studies were not designed for examining antimicrobial resistance or risk factors associated with resistance. Therefore, several variables were not collected that would have been important when identifying risk factors associated with antimicrobial resistance, such as recent hospital visits, other healthcare exposures, water and sanitation conditions at homes, or crowding. Also, information on risk factors such as HIV infection was not available for every patient across the three studies.

Injudicious antimicrobial use in the human and agriculture sectors are among the major factors contributing to antimicrobial resistance worldwide [23]. Widespread and unregulated use of antimicrobials is common in many countries, including Kenya. The findings from our survey on antimicrobial use identified the same issues related to misuse of antimicrobials for humans and animals; ceftriaxone and other antibiotics, to which NTS isolates were resistant, were widely prescribed and purchased even without requiring doctor’s or veterinarian’s prescriptions. The report of a situation analysis conducted in Kenya in 2011 similarly noted antibiotic misuse by patients, doctors, and other licensed prescribers as a factor affecting antibiotic resistance in Kenya and listed lack of diagnostic capacity, fear of negative outcomes if antibiotics are withheld, limited access to healthcare services, and self-medication as possible reasons for misuse [24].

In addition to the risk factors we analyzed, the rapid emergence of ceftriaxone-resistant NTS in Kenya may be driven by the bigger epidemic in sub-Saharan Africa. Clonal expansion of multidrug-resistant *Salmonella* Typhimurium strain of sequence type ST313 has played a major role in the dissemination of multidrug-resistant NTS in Africa [25]. *Salmonella* Typhimurium ST313 strains have been identified in all 4 isolates tested for the current study, and in previous studies conducted at a private hospital in Nairobi [16, 18].

In summary, ceftriaxone-resistant serotype *Salmonella* Typhimurium infections, which were rarely reported in western Kenya, rapidly emerged in 2009–2014, limiting treatment options for serious infections. Efforts are ongoing to address the urgent need for improved microbiologic diagnostic capacity in clinical settings to guide appropriate treatment in rural Kenya [26]. In the long run, development of a surveillance system to monitor antimicrobial resistance and measures to better control antibiotic misuse in human and agriculture sectors in Kenya will be important for detecting and responding to emerging resistance and for preserving the effectiveness of antimicrobials.

## Supporting information

S1 Appendix Questionnaire for clinicians/nurses. (DOCX)

S2 Appendix Questionnaire for pharmacies. (DOCX)

S3 Appendix Questionnaire for agrovets. (DOCX)

S1 Dataset (DOCX)

S1 Table Results of antimicrobial use survey conducted among health care-providers in Siaya county, Kenya, 2014. (DOCX)

S2 Table Results of antimicrobial use survey conducted among pharmacies in Siaya county, Kenya, 2014. (DOCX)

S3 Table Results of antimicrobial use survey conducted among agrovets or shops that supply drugs, feeds, and other products for livestock, Siaya county, Kenya, 2014. (DOCX)

S4 Table Serotyping and whole genome sequencing results of 4 selected non-typhoidal *Salmonella* isolates from invasive infections identified among central nervous system infection study (CNS study), and International Emerging Infections Program (surveillance program) participants during 2009–2014, Siaya county, Kenya. (DOCX)

## References

[1] Feasey NA, Dougan G, Kingsley RA, Heyderman RS, Gordon MA. Invasive non-typhoidal salmonella disease: an emerging and neglected tropical disease in Africa. Lancet. 2012;379(9835):2489–99. 10.1016/S0140-6736(11)61752-2. . 22587967 PMC3402672

[2] Marks F, von Kalckreuth V, Aaby P, Adu-Sarkodie Y, El Tayeb MA, Ali M, et al Incidence of invasive salmonella disease in sub-Saharan Africa: a multicentre population-based surveillance study. The Lancet Global health. 2017;5(3):e310–e23. Epub 2017/02/15. 10.1016/S2214-109X(17)30022-0 . 28193398 PMC5316558

[3] Morpeth SC, Ramadhani HO, Crump JA. Invasive non-Typhi Salmonella disease in Africa. Clinical infectious diseases: an official publication of the Infectious Diseases Society of America. 2009;49(4):606–11. Epub 2009/07/14. 10.1086/603553 . 19591599 PMC2741563

[4] Uche IV, MacLennan CA, Saul A. A Systematic Review of the Incidence, Risk Factors and Case Fatality Rates of Invasive Nontyphoidal Salmonella (iNTS) Disease in Africa (1966 to 2014). PLoS neglected tropical diseases. 2017;11(1):e0005118 Epub 2017/01/06. 10.1371/journal.pntd.0005118 . 28056035 PMC5215826

[5] Reddy EA, Shaw AV, Crump JA. Community-acquired bloodstream infections in Africa: a systematic review and meta-analysis. The Lancet infectious diseases. 2010;10(6):417–32. Epub 2010/06/01. 10.1016/S1473-3099(10)70072-4 . 20510282 PMC3168734

[6] Verani JR, Toroitich S, Auko J, Kiplang'at S, Cosmas L, Audi A, et al Burden of Invasive Nontyphoidal Salmonella Disease in a Rural and Urban Site in Kenya, 2009–2014. Clinical infectious diseases: an official publication of the Infectious Diseases Society of America. 2015;61 Suppl 4:S302–9. 10.1093/cid/civ728 . 26449945 PMC4800067

[7] Mahon BE, Fields PI. Invasive Infections with Nontyphoidal Salmonella in Sub-Saharan Africa. Microbiol Spectr. 2016;4(3). Epub 2016/06/24. 10.1128/microbiolspec.EI10-0015-2016 . 27337467

[8] Crump JA, Sjolund-Karlsson M, Gordon MA, Parry CM. Epidemiology, Clinical Presentation, Laboratory Diagnosis, Antimicrobial Resistance, and Antimicrobial Management of Invasive Salmonella Infections. Clinical microbiology reviews. 2015;28(4):901–37. 10.1128/CMR.00002-15 . 26180063 PMC4503790

[9] Oneko M, Kariuki S, Muturi-Kioi V, Otieno K, Otieno VO, Williamson JM, et al Emergence of Community-Acquired, Multidrug-Resistant Invasive Nontyphoidal Salmonella Disease in Rural Western Kenya, 2009–2013. Clinical infectious diseases: an official publication of the Infectious Diseases Society of America. 2015;61 Suppl 4:S310–6. 10.1093/cid/civ674 . 26449946

[10] Onyango CO, Loparev V, Lidechi S, Bhullar V, Schmid DS, Radford K, et al Evaluation of a TaqMan Array Card for Detection of Central Nervous System Infections. Journal of clinical microbiology. 2017;55(7):2035–44. Epub 2017/04/14. 10.1128/JCM.02469-16 . 28404679 PMC5483905

[11] Feikin DR, Audi A, Olack B, Bigogo GM, Polyak C, Burke H, et al Evaluation of the optimal recall period for disease symptoms in home-based morbidity surveillance in rural and urban Kenya. International journal of epidemiology. 2010;39(2):450–8. 10.1093/ije/dyp374 . 20089695 PMC2846445

[12] CLSI. Performance Standards for Antimicrobial Susceptibility Testing; Twenty-Seventh Informational Supplement. CLSI document M100-S27. Wayne, PA: Clinical and Laboratory Standards Institute; 2017.

[13] Strockbine NA B C, Fields PI, Kaper JB, and Natoro JP. Escherichia, Shigella, and Salmonella In: Versalovic J, Carroll K, Funke G, Jorgensen J, Landry M, Warnock D, editor. Manual of Clinical Microbiology. Washington D.C: ASM Press; 2015 p. 685–713.

[14] Tagg KA, Francois Watkins L, Moore MD, Bennett C, Joung YJ, Chen JC, et al Novel trimethoprim resistance gene dfrA34 identified in Salmonella Heidelberg in the USA. The Journal of antimicrobial chemotherapy. 2019;74(1):38–41. Epub 2018/09/12. 10.1093/jac/dky373 . 30202900 PMC10870229

[15] Odhiambo F, Galgalo T, Wences A, Muchemi OM, Kanyina EW, Tonui JC, et al Antimicrobial resistance: capacity and practices among clinical laboratories in Kenya, 2013. The Pan African medical journal. 2014;19:332 Epub 2014/01/01. 10.11604/pamj.2014.19.332.5159 . 25918572 PMC4405071

[16] Kariuki S, Onsare RS. Epidemiology and Genomics of Invasive Nontyphoidal Salmonella Infections in Kenya. Clinical infectious diseases: an official publication of the Infectious Diseases Society of America. 2015;61 Suppl 4:S317–24. 10.1093/cid/civ711 . 26449947 PMC4596933

[17] Okoth C, Opanga S, Okalebo F, Oluka M, Baker Kurdi A, Godman B. Point prevalence survey of antibiotic use and resistance at a referral hospital in Kenya: findings and implications. Hosp Pract (1995). 2018;46(3):128–36. Epub 2018/04/14. 10.1080/21548331.2018.1464872 . 29652559

[18] Kariuki S, Okoro C, Kiiru J, Njoroge S, Omuse G, Langridge G, et al Ceftriaxone-resistant Salmonella enterica serotype typhimurium sequence type 313 from Kenyan patients is associated with the blaCTX-M-15 gene on a novel IncHI2 plasmid. Antimicrobial agents and chemotherapy. 2015;59(6):3133–9. 10.1128/AAC.00078-15 . 25779570 PMC4432211

[19] Kenya MoH. National Manual for the management of HIV-related opportunistic infections and conditions. 1st ed Kenya: BALTECH EQUIPMENTS LTD, Nairobi; 2008.

[20] Arcilla MS, van Hattem JM, Haverkate MR, Bootsma MCJ, van Genderen PJJ, Goorhuis A, et al Import and spread of extended-spectrum beta-lactamase-producing Enterobacteriaceae by international travellers (COMBAT study): a prospective, multicentre cohort study. The Lancet infectious diseases. 2017;17(1):78–85. 10.1016/S1473-3099(16)30319-X . 27751772

[21] van den Bunt G, Liakopoulos A, Mevius DJ, Geurts Y, Fluit AC, Bonten MJ, et al ESBL/AmpC-producing Enterobacteriaceae in households with children of preschool age: prevalence, risk factors and co-carriage. The Journal of antimicrobial chemotherapy. 2017;72(2):589–95. 10.1093/jac/dkw443 . 27789683

[22] Liakopoulos A, van den Bunt G, Geurts Y, Bootsma MCJ, Toleman M, Ceccarelli D, et al High Prevalence of Intra-Familial Co-colonization by Extended-Spectrum Cephalosporin Resistant Enterobacteriaceae in Preschool Children and Their Parents in Dutch Households. Frontiers in microbiology. 2018;9:293 10.3389/fmicb.2018.00293 . 29515562 PMC5826366

[23] Laxminarayan R, Duse A, Wattal C, Zaidi AK, Wertheim HF, Sumpradit N, et al Antibiotic resistance-the need for global solutions. The Lancet infectious diseases. 2013;13(12):1057–98. Epub 2013/11/21. 10.1016/S1473-3099(13)70318-9 . 24252483

[24] Global Antibiotic Resistance Partnership-Kenya Working Group. Situation Analysis and Recommendations: Antibiotic Use and Resistance in Kenya. Washington, DC and New Delhi: Center for Disease Dynamics, Economics & Policy, 2011.

[25] Okoro CK, Kingsley RA, Connor TR, Harris SR, Parry CM, Al-Mashhadani MN, et al Intracontinental spread of human invasive Salmonella Typhimurium pathovariants in sub-Saharan Africa. Nat Genet. 2012;44(11):1215–21. 10.1038/ng.2423. . 23023330 PMC3491877

[26] Hunsperger E, Juma B, Onyango C, Ochieng JB, Omballa V, Fields BS, et al Building laboratory capacity to detect and characterize pathogens of public and global health security concern in Kenya. BMC public health. 2019;19(3):477 10.1186/s12889-019-6770-9 PMC6696698 32326916

