# Supplementary material for: Exploration of risk factors for ceftriaxone resistance in invasive non-typhoidal Salmonella infections in western Kenya
Source: PLoS One. 2020 Mar 3;15(3):e0229581. doi: 10.1371/journal.pone.0229581 (PMC7053705; doi:10.1371/journal.pone.0229581)
Supplement: S1 Appendix — (DOCX) [file pone.0229581.s001.docx]

## S1 Appendix. Questionnaire for Clinicians/Nurses

**ID Number:** __________ **Date:** ___/___/___ **Interviewer:** ________

**Clinician/Nurse Questionnaire**

We’re working with the Centers for Disease Control and Prevention in USA and Ministry of Health on a study of antibiotic use both in human and in animals in this area. My name is [insert name] and these are my colleagues [insert names]. We are talking with several hospitals as well as pharmacies and agrovets in this area and would like to ask for about 10 minutes of your time to ask some questions about your opinions and experiences with antibiotics. We will not be recording your name, and your participation is voluntary. If you have any questions, I would be happy to answer them.

**PART 1 – GENERAL INFORMATION – TO BE COMPLETED FOR EVERY HOSPITAL**

**Hospital: _________________________________________________**

**Person/s interviewed:** □ Clinician □ Nurse □ Other (specify)**:** ______________________________

**Hospital type:** □ County referral □ Sub-county □ Mission □ Private □ Other (specify)**:** _____________

**PART 2 – ANTIBIOTIC INFORMATION**

1. **Does your ward prescribe any 3^rd^ generation cephalosporins such as ceftriaxone (e.g. Rocephin, Ceftrimet), ceftazidime (e.g. C Zid), cefexime or cefotaxime?**

□ Yes □ No [skip to question 13] □ Don’t know [skip to question 13]

1. **Could you please name the 3^rd^ generation cephalosporins that are prescribed most often at your ward?** (List them)

______________________________________________________________________________

______________________________________________________________________________

1. **When did they first become available at your hospital?** (Write down the year/s)______________________
2. **In your opinion, what is the trend in prescription of the 3^rd^ generation cephalosporins since they became first available at your hospital?**

□ Increasing □ Decreasing □ Didn’t change □ Don’t know

1. **How often did your clinicians prescribe them for children with below conditions during the past year? As I read the conditions, please choose between Always, Often, Sometimes, Rarely and Never.**

| **Conditions** | **Always** | **Often** | **Sometimes** | **Rarely** | **Never** |
| --- | --- | --- | --- | --- | --- |
| Admitted with acute febrile illness | □ | □ | □ | □ | □ |
| Admitted with suspected sepsis | □ | □ | □ | □ | □ |
| Admitted with suspected meningitis | □ | □ | □ | □ | □ |
| Any other | □ | □ | □ | □ | □ |

1. **Which of the commonly prescribed 3^rd^ generation cephalosporins that you mentioned above are available in your ward today?** (List them)

______________________________________________________________________________

______________________________________________________________________________

1. **During the past year, how often have the commonly prescribed 3^rd^ generation cephalosporins not been available at your hospital?**

□ Always □ Often □ Sometimes □ Rarely □ Never [skip to question 10]

1. **In the past 30 days, on how many days do you think ceftriaxone or other 3^rd^ generation cephalosporins were not available at your hospital?** **_______________**
2. **When they become unavailable at your hospital, where did the inpatients buy their prescribed drugs?** (mark all that apply)

□ Registered pharmacy □ Unregistered pharmacy □ Non-pharmacy stores

□ Others (list)­­­ ­­­ __________________________________________________________________________

1. **How often do the families of admitted patients have situations when they cannot buy a full dose of the prescribed 3^rd^ generation cephalosporins?**

□ Always □ Often □ Sometimes □ Rarely □ Never [skip to question 12]

1. **If the families of admitted patients cannot afford to buy the full prescribed 3^rd^ generation cephalosporin dose, what do they commonly do?**

□ Don’t purchase any antibiotics

□ Purchase a different antibiotic that is more affordable

□ Purchase a partial dose then purchase the remainder of the prescribed dose later when they can afford it

□ Other (specify) ­­­­­­__________________________________________________________________________

1. **How often do you think the patients buy the 3^rd^ generation cephalosporins without a clinician’s prescription to self-treat or following other people’s recommendations?**

□ Always □ Often □ Sometimes □ Rarely □ Never

1. **In the past year, has your hospital prescribed other cephalosporins such as the 1^st^ generation cephalosporin–cefazolin and the 2^nd^ generation cephalosporin-cefuroxime (e.g. Zinnat)?**

□ Yes □ No [If no, skip to question 16] □ Don’t know [If no, skip to question 16]

1. **What brands of 1^st^ and 2^nd^ generation cephalosporins (e.g. Cefalexin and Zinnat) are available at your hospital today?** (List them)

______________________________________________________________________________

______________________________________________________________________________

1. **During the past year, how often would above mentioned 1^st^ and 2^nd^ generation cephalosporins been not available in your hospital?**

□ Always □ Often □ Sometimes □ Rarely □ Never

1. **In the past year, has your hospital prescribed below antibiotics and are they available at your hospital today? I will read the antibiotic names and you can choose the answers Yes, No, and Don’t Know.**

| **Antibiotics** | **In the past year, has it been prescribed for children admitted to your ward?** | | | **Is it available for children today?** | | |
| --- | --- | --- | --- | --- | --- | --- |
|  | **Yes** | **No** | **DK** | **Yes** | **No** | **DK** |
| Other beta-lactam antibiotics, such as amoxicillin and penicillin | □ | □ | □ | □ | □ | □ |
| Ciprofloxacin | □ | □ | □ | □ | □ | □ |
| Co-trimoxazole (e.g. Septrin) | □ | □ | □ | □ | □ | □ |
| Gentamicin | □ | □ | □ | □ | □ | □ |

1. **During the past year, how often would below drugs become unavailable in your hospital? I will read the antibiotic names and you can choose the answers Always, Often, Sometimes, Raraly and Never.**

| **Antibiotics** | **Always** | **Often** | **Sometimes** | **Rarely** | **Never** |
| --- | --- | --- | --- | --- | --- |
| Other beta-lactam antibiotics, such as amoxicillin and penicillin | □ | □ | □ | □ | □ |
| Ciprofloxacin | □ | □ | □ | □ | □ |
| Co-trimoxazole (e.g. Septrin) | □ | □ | □ | □ | □ |
| Gentamicin | □ | □ | □ | □ | □ |

1. **In the past year, have carbapenems such as meropenem and imipenem been available at your hospital?**

□ Yes □ No [If no, skip to question 20] □ Don’t know

1. **Are carbapenems such as meropenem and imipenem available at your hospital today?**

□ Yes □ No □ Don’t know

1. **How often do your clinicians prescribe carbapenems for the patient’s family to purchase it?**

□ Always □ Often □ Sometimes □ Rarely □ Never

**20. In your opinion, what is the most common way by which people here encounter antimicrobial resistance?**

______________________________________________________________________________

______________________________________________________________________________

______________________________________________________________________________

**That’s all of my questions. Is there anything else you think I should know about antibiotics available at your hospital?**

______________________________________________________________________________

______________________________________________________________________________

______________________________________________________________________________

______________________________________________________________________________

**Thank you for your time and participation!**
