# Supplementary material for: Exploration of risk factors for ceftriaxone resistance in invasive non-typhoidal Salmonella infections in western Kenya
Source: PLoS One. 2020 Mar 3;15(3):e0229581. doi: 10.1371/journal.pone.0229581 (PMC7053705; doi:10.1371/journal.pone.0229581)
Supplement: S2 Appendix — (DOCX) [file pone.0229581.s002.docx]

## S2 Appendix. Questionnaire for Pharmacies

**ID Number:**____________ **Date:** ___/___/___ **Interviewer:** ______

**Pharmacy Questionnaire**

[Ask for the owner or senior staff person at the facility.]

We’re working with the Centers for Disease Control and Prevention in USA and Ministry of Health on a study of antibiotic use both in human and in animals in this area. My name is [insert name] and these are my colleagues [insert names]. We are talking with several pharmacies as well as hospitals and agrovets in this area and would like to ask for about 10 minutes of your time to ask some questions about your opinions and experiences with antibiotics. We will not be recording your name, and your participation is voluntary. If you have any questions, I would be happy to answer them.

**PART 1 – GENERAL INFORMATION – TO BE COMPLETED FOR EVERY PERSON INTERVIEWED**

**Person interviewed:** □ Owner □ Employee □ Other (specify)**:** ____________________

***If hospital pharmacy:** □ Pharmacist □ Pharm Tech □ Other (specify**):** ____________________

**Facility type:** □ Hospital □ General Pharmacy □ Kiosk □ Other (specify)**:** ____________________

**Distance from hospital:** □ Less than 1 km □ 1-5 km □ Greater than 5 km

**PART 2 – ANTIBIOTIC INFORMATION**

1. **Do you dispense antibiotics?**

□ Yes □ No [If no, end of interview] □ Don’t know

1. **In the past year, has your pharmacy stocked any 3^rd^ generation cephalosporins, such as ceftriaxone (e.g., Rocephin, Ceftrimet), ceftazidime (e.g., C Zid), cefotaxime, and cefixime?**

□ Yes □ No [If no, skip to question 7] □ Don’t know

1. **What 3^rd^ generation cephalosporins do you have in stock?** _______________________
2. **What brands are available for purchase today?** ________________________________
   1. **May we see the packaging for these antibiotics to confirm the spelling of the brand name?**

1. **What year did 3^rd^ generation cephalosporins first become available in your pharmacy?** ____________________
2. **During the past year, how often have 3^rd^ generation cephalosporins been unavailable in your pharmacy?**

□ Always □ Often □ Sometimes □ Rarely □ Never

1. **In the past year, has your pharmacy stocked any other cephalosporins (e.g., 1^st^ generation: cephalothin; 2^nd^ generation: cefoxitin)?**

□ Yes □ No [If no, skip to question 10] □ Don’t know

1. **What brands are available for purchase today?** ________________________________
   1. **May we see the packaging for these antibiotics to confirm the spelling of the brand name?**
2. **During the past year, how often would other cephalosporins become unavailable in your pharmacy?**

□ Always □ Often □ Sometimes □ Rarely □ Never

1. **In the past year, has your pharmacy stocked any other beta-lactam antibiotics, such as amoxicillin, penicillins, or any others?**

□ Yes □ No [If no, skip to question 14] □ Don’t know

1. **Are other beta-lactam antibiotics (e.g., amoxicillin, penicillin) available for purchase today?**

□ Yes □ No [If no, skip to question 13] □ Don’t know

1. **What other beta-lactam antibiotics are available today?** ____________________
2. **During the past year, how often have other beta-lactam antibiotics been unavailable in your pharmacy?**

□ Always □ Often □ Sometimes □ Rarely □ Never

1. **In the past year, has your pharmacy stocked ciprofloxacin (e.g., Abact)?**

□ Yes □ No [If no, skip to question 17] □ Don’t know

1. **Is ciprofloxacin (e.g., Abact) available for purchase today?**

□ Yes □ No □ Don’t know

1. **During the past year, how often would ciprofloxacin (e.g., Abact) become unavailable in your pharmacy?**

□ Always □ Often □ Sometimes □ Rarely □ Never

1. **In the past year, has your pharmacy stocked co-trimoxazole (e.g., Septrin)?**

□ Yes □ No [If no, skip to question 20] □ Don’t know

1. **Is co-trimoxazole (e.g., Septrin) available for purchase today?**

□ Yes □ No □ Don’t know

1. **During the past year, how often would co-trimoxazole (e.g., Septrin) become unavailable in your pharmacy?**

□ Always □ Often □ Sometimes □ Rarely □ Never

1. **In the past year, has your pharmacy stocked gentamicin?**

□ Yes □ No [If no, skip to question 23] □ Don’t know

1. **Is gentamicin available for purchase today?**

□ Yes □ No □ Don’t know

1. **During the past year, how often would gentamicin become unavailable in your pharmacy?**

□ Always □ Often □ Sometimes □ Rarely □ Never

1. **In the past year, has your pharmacy stocked any Fansidar (i.e., sulfadoxine-pyrimethamine)?**

□ Yes □ No [If no, skip to question 26] □ Don’t know

1. **Is Fansidar available for purchase today?**

□ Yes □ No □ Don’t know

1. **During the past year, how often would Fansidar become unavailable in your pharmacy?**

□ Always □ Often □ Sometimes □ Rarely □ Never

1. **In the past year, have carbapenems (e.g., Meropenem, Imipenem) ever been available for purchase in your store/hospital?**

□ Yes □ No [If no, skip to question 29] □ Don’t know

1. **Are carbapenems (e.g.,** **Meropenem, Imipenem) available for purchase now?**

□ Yes □ No □ Don’t know

1. **Have patients ever asked to purchase carbapenems (e.g., Meropenem, Imipenem)?**

□ Yes □ No □ Don’t know

1. **A patient may not always be able to purchase a full prescribed course of antibiotics. In your experience, how often does this happen?**

□ Always □ Often □ Sometimes □ Rarely □ Never [If never, end interview]

1. **If such a patient requests to purchase a partial prescription, would you sell it?**

□ Yes □ No [If no, skip to question 32]

1. **If yes, does the patient return to purchase the rest of the prescription?**

□ Always □ Often □ Sometimes □ Rarely □ Never

1. **That’s all of the questions we have. Is there anything else you would like to tell me about antibiotics at your shop or in this area?**

______________________________________________________________________________

______________________________________________________________________________

______________________________________________________________________________

______________________________________________________________________________

*Thank you for your time!*

1. **Was a pharmacy license observed in the store or did the respondent mention that the pharmacy is licensed?**

□ Yes—registered □ No—may not be registered
