# Supplementary material for: Exploration of risk factors for ceftriaxone resistance in invasive non-typhoidal Salmonella infections in western Kenya
Source: PLoS One. 2020 Mar 3;15(3):e0229581. doi: 10.1371/journal.pone.0229581 (PMC7053705; doi:10.1371/journal.pone.0229581)
Supplement: S3 Appendix — (DOCX) [file pone.0229581.s003.docx]

## S3 Appendix. Questionnaire for Agrovets

**ID Number:** ____________________ **Date:** ___/___/___ **Interviewer:** ________

**Agrovet Questionnaire**

[Ask for the owner or senior staff person at the shop.]

We’re working with the Centers for Disease Control and Prevention in USA and Ministry of Health on a study of antibiotic use both in human and in animals in this area. My name is [insert name] and these are my colleagues [names]. We are talking with several pharmacies, hospitals and Agrovets in this area and would like to ask for about 10 minutes of your time to ask some questions about your opinions and experiences with antibiotics. We will not be recording your name or the name or location of your shop and your participation is voluntary. If you have any questions, I would be happy to answer them.

**PART 1 – GENERAL INFORMATION – TO BE COMPLETED FOR EVERY PERSON INTERVIEWED**

**Person interviewed:** □ Owner □ Employee □ Other (specify)**:** _____________

**PART 2 – ANTIBIOTIC INFORMATION**

1. **Do you sell animal health products?**

□ Yes □ No (If no, end of the questionnaire) □ Don’t know

1. **Do you sell antibiotics for use in animals?**

□ Yes □ No (If no, skip to Q8) □ Don’t know

1. **Do you sell the following classes of antibiotics?**

| **Antibiotic** | **Yes** | **No** | **DK** | **Livestock species** | | | | |
| --- | --- | --- | --- | --- | --- | --- | --- | --- |
|  |  |  |  | **Cattle** | **Sheep** | **Goats** | **Poultry** | **Other** |
| Beta lactams [penicillin, ampicillin (e.g Betamox®, Ampiclox)] |  |  |  |  |  |  |  |  |
| Aminoglycoside (Gentamicin e.g Gentamast®) |  |  |  |  |  |  |  |  |
| Tetracyclines (Oxytetracycline) |  |  |  |  |  |  |  |  |
| Quinolones [Flouroquinole (e.g Meriquine ®)] |  |  |  |  |  |  |  |  |
| Macrolides (Tylosin e.g Fosbac®, Erythromycin e.g Aliseryl®) |  |  |  |  |  |  |  |  |
| Sulphonamides ( sulphadimidine e.g Biotrim®, S-Dime) |  |  |  |  |  |  |  |  |

1. **What is the main reason for farmers to purchase antibiotics?**

□ Treatment □ Prophylaxis □ Growth promotion □ Don’t know □ Others**________________________**

1. **Do you sell cephalosporin antibiotics such as cefalexine (e.g Solvasol®)?**

□ Yes □ No □ Don’t know

1. **Which livestock species is it used for?**

□ Cattle □ Sheep □ Goat □ Poultry □ Others **________**

1. **When did Cephalosporin antibiotics become first available at your agrovet?(record the year)**

**________**

1. **Do you sell animal feeds?** □ Yes □ No (If no, skip to Q 13) □ Don’t know
2. **Do you sell poultry feeds?**  □ Yes □ No (If no, skip to Q 11) □ Don’t know
3. **Poultry feeds sold in the Agrovet**

| Can you please allow me to see the package for the three most commonly sold poultry feeds? | As far as you know, does this poultry feed have any additive/ antibiotic in it? (Y/N) | Interviewer observation of antibiotic content of the feed, if any (list the antibiotics if any on the package) |
| --- | --- | --- |
| Brand name 1: |  |  |
| Brand name 2: |  |  |
| Brand name 3: |  |  |

1. **Do you sell cattle, sheep and goats feeds?**

□ Yes □ No (If no, skip to Q 13) □ Don’t know

1. **Cattle, sheep and goats feeds sold in the Agrovet**

| Can you please allow me to see the package for the three most commonly sold cattle, sheep and goats feed? | As far as you know, does this feed have any additive/ antibiotic in it? (Y/N) | Interviewer observation of antibiotic content of the feed, if any (list the antibiotics if any on the package) |
| --- | --- | --- |
| Brand name 1: |  |  |
| Brand name 2: |  |  |
| Brand name 3: |  |  |

1. **Do you sell animal supplements (e.g soluble multivitamins)?**

□ Yes □ No (If no, end of the questionnaire) □ Don’t know

1. **Do you sell poultry supplements?**

□ Yes □ No (If no, skip to Q 16) □Don’t know

1. **Poultry supplements sold in the Agrovet**

| Can you please allow me to see the package for the three most commonly sold poultry supplements? | As far as you know, does this poultry supplement have any antibiotic in it? (Y/N) | Interviewer observation of antibiotic content of the supplement, if any (list the antibiotics if any on the package) |
| --- | --- | --- |
| Brand name 1: |  |  |
| Brand name 2: |  |  |
| Brand name 3: |  |  |

1. **Do you sell cattle, sheep and goat supplements?**

□ Yes □ No (If no, end the questionnaire) □ Don’t know

1. **Cattle, sheep and goats supplements sold in the Agrovet**

| Can you please allow me to see the package for the three most commonly sold cattle and sheep and goats supplements? | As far as you know, does this supplement have any antibiotic in it? (Y/N) | Interviewer observation of antibiotic content of the supplement, if any (list the antibiotics if any on the package) |
| --- | --- | --- |
| Brand name 1: |  |  |
| Brand name 2: |  |  |
| Brand name 3: |  |  |

**That’s all of the questions we have. Is there anything else you would like to tell me about antibiotics at your shop or in this area?**

______________________________________________________________________________

______________________________________________________________________________

Thank you very much for your time and agreeing to be interviewed.

License (*observed in store or mentioned by respondent*): □ Yes □ No
