# Supplementary material for: Exploration of risk factors for ceftriaxone resistance in invasive non-typhoidal Salmonella infections in western Kenya
Source: PLoS One. 2020 Mar 3;15(3):e0229581. doi: 10.1371/journal.pone.0229581 (PMC7053705; doi:10.1371/journal.pone.0229581)
Supplement: S1 Dataset — (DOCX) [file pone.0229581.s004.docx]

**Data 1. Patients with invasive non-typhoidal *Salmonella* infections among malaria vaccine trial participants during 2009–2013, Siaya county, Kenya.**

| **Variable** | **Invasive non-typhoidal *Salmonella*** | | | |
| --- | --- | --- | --- | --- |
|  | **Ceftriaxone-resistant**  **N=16** | | **Ceftriaxone-susceptible**  **N=74** | |
| Age in months median (range)^ⱡ^ | 35 | (4–53) | 15 | (2–47) |
| Male gender | 6 | (38%) | 35 | (47%) |
| HIV positive^#^ | 2 | (13%) | 7 | (10%) |
| Has HIV-positive mother | 9 | (56%) | 19 | (26%) |
| Has malaria at the time of invasive non-typhoidal *Salmonella* disease diagnosis | 8 | (50%) | 37 | (50%) |
| Had malaria within 2 weeks before invasive NTS disease diagnosis | 10 | (63%) | 46 | (62%) |
| Died | 0 | (0%) | 3 | (4%) |

^ⱡ^The older age of ceftriaxone-resistant patients was related to the longitudinal nature of the study, and the emergence of resistance relatively late in the study

^#^Missing data were excluded from the analyses

**Data 2. Patients with invasive non-typhoidal *Salmonella* infections among central nervous system infection study participants during 2012–2013, Siaya county, Kenya.**

| **Variable** | **Invasive non-typhoidal *Salmonella*** | | | |
| --- | --- | --- | --- | --- |
|  | **Ceftriaxone-resistant**  **N=16** | | **Ceftriaxone-susceptible**  **N=74** | |
| Age in months, median (range) | 16 | (3-62) | 15 | (2–28) |
| Male gender | 10 | (63%) | 0/2 | (0) |
| Seeking healthcare because child is sick (vs. retuning for for Tx, immunization etc.) | 11 | (73%) | 1/2 | (50%) |
| Took antibiotics before arriving to hospital^#^ | 5 | (56%) | 1 | (100%) |
| Sought healthcare before arriving to this hospital | 8 | (72%) | 2 | (100%) |
| Fever^#^ | 14 | (88%) | 2 | (100%) |
| Temperature in Celsius, median (range) ^#^ | 38 | (36–40) | 39 | (39–39) |
| Stiff neck^#^ | 7 | (47%) | 1 | (50%) |
| Diarrhea^#^ | 6 | (43%) | 2 | (100%) |

^#^Missing data were excluded from the analyses

**Data 3. Data of patients with invasive non-typhoidal *Salmonella* infections among International Emerging Infections Program participants during 2009–2014, Siaya county, Kenya.**

| **Variable** | **Invasive non-typhoidal *Salmonella*** | | | |
| --- | --- | --- | --- | --- |
|  | **Ceftriaxone-resistant**  **N=11** | | **Ceftriaxone-susceptible**  **N=185** | |
| Sought some kind of healthcare before presenting to the clinic^#^ | 5 | (46%) | 95 | (52%) |
| Taken a medication for this illness | 5 | (46%) | 97 | (52%) |
| Sulfadoxine/pyrimethamine | 0 | (0%) | 2 | (1%) |
| Septrin | 2 | (18%) | 7 | (4%) |
| Penicillin | 0 | (0%) | 9 | (5%) |
| Other antimicrobials | 0 | (0%) | 3 | (2%) |
| Examination |  |  |  |  |
| Temperature: median (range) | 39.3 | (36.7–40.4) | 38.8 | (35.0–41.0) |
| Had fever^#^ | 11 | (100%) | 175 | (95%) |
| Days with fever (median,  range) | 3 (1–4) | | 3 (0–61) | |
| Had diarrhea^#^ | 0 | (0%) | 47 | (26%) |
| Vomited^#^ | 1 | (9%) | 51 | (28%) |
| Initial diagnosis-pneumonia^#^ | 2 | (18%) | 17 | (9%) |
| Initial diagnosis-diarrhea^#^ | 0 | (0.0%) | 36 | (20%) |
| Participant taken/given antibiotics on the day of interview ^#^ | 5 | (45%) | 137 | (75%) |
| septrin^#^ | 0 | (0%) | 29 | (22%) |
| gentamicin^#^ | 1 | (20%) | 21 | (15%) |
| ciprofloxacin^#^ | 0 | (0%) | 12 | (9%) |
| erythromycin^#^ | 1 | (20%) | 21 | (15%) |
| chloramphenicol^#^ | 0 | (0%) | 6 | (4%) |
| amoxicillin/ampicillin^#^ | 1 | (20%) | 31 | (23%) |
| tetracycline^#^ | 0 | (0%) | 2 | (2%) |
| penicillin^#^ | 1 | (20%) | 25 | (18%) |
| metronidazole^#^ | 0 | (0%) | 19 | (14%) |
| ceftriaxone^#^ | 0 | (0%) | 8 | (6%) |
| Antibiotic given during the hospital course^#^ | 1 | (33%) | 47 | (98%) |
| septrin^#^ | 0 | (0%) | 12 | (26%) |
| gentamicin^#^ | 1 | (100%) | 18 | (38%) |
| ciprofloxacin^#^ | 0 | (0%) | 2 | (4%) |
| erythromycin^#^ | 0 | (0%) | 5 | (11%) |
| chloramphenicol^#^ | 0 | (0%) | 1 | (2%) |
| amoxicillin/ampicillin^#^ | 0 | (0%) | 11 | (23%) |
| tetracycline^#^ | 0 | (0%) | 1 | (2%) |
| nalidixic acid^#^ | 0 | (0%) | 6 | (13%) |
| penicillin^#^ | 1 | (100%) | 19 | (40%) |
| metronidazole^#^ | 0 | (0%) | 11 | (23%) |
| ceftriaxone^#^ | 1 | (100%) | 11 | (23%) |
| Admitted to Hospital^#^ | 5 | (46%) | 52 | (28%) |
| Duration of hospitalization,  days (median, range) | 3 (2–7) | | 3 (0–9) | |
| Discharge diagnosis-malaria | 3 | (60%) | 14 | (27%) |
| Discharge diagnosis-diarrhea | 0 | (0%) | 20 | (39%) |
| Discharged without sequelae | 4 | (100%) | 49 | (94%) |

^#^Missing data were excluded from the analyses

**Data 4**. **Antibiotic susceptibility test results of the** **non-typhoidal *Salmonella* isolates from invasive infections identified among malaria vaccine trial (vaccine trial), central nervous system infection study (CNS study), and International Emerging Infections Program (surveillance program) participants during 2009–2014, Siaya county, Kenya**

|  | **Vaccine trial** | | | | | | **CNS study** | | | | | | **Surveillance program** | | | | | |
| --- | --- | --- | --- | --- | --- | --- | --- | --- | --- | --- | --- | --- | --- | --- | --- | --- | --- | --- |
|  | Ceftriaxone-R | | | Cef-S | | | Ceftriaxone-R | | | Cef-S | | | Ceftriaxone-R | | | Cef-S | | |
| **Antimicrobial** | n resistant | n tested | % | n resistant | n tested | % | n resistant | n tested | % | n resistant | n tested | % | n resistant | n tested | % | n resistant | n tested | % |
| Gentamicin | 16 | 16 | 100% | 0 | 74 | 0% | 16 | 16 | 100% | 0 | 2 | 0% | 9 | 11 | 82% | 5 | 154 | 3% |
| Kanamycin | 14 | 15 | 93% | 1 | 74 | 1% | 16 | 16 | 100% | 0 | 0 | 0% | 9 | 11 | 82% | 11 | 154 | 7% |
| Streptomycin | 6 | 6 | 100% | 59 | 64 | 92% | 15 | 16 | 94% | 2 | 2 | 100% | 11 | 11 | 100% | 138 | 155 | 89% |
| Amoxicillin-clavulanic acid | 16 | 16 | 100% | 34 | 66 | 52% | 16 | 16 | 100% | 1 | 2 | 50% | 6 | 10 | 60% | 62 | 146 | 42% |
| Cefotaxime | 1 | 1 | 100% | 0 | 1 | 0% | 0 | 0 |  | 0 | 0 |  | 0 |  |  | 0 |  |  |
| Sulfisoxazole | 13 | 13 | 100% | 34 | 40 | 85% | 16 | 16 | 100% | 2 | 2 | 100% | 11 | 11 | 100% | 140 | 154 | 91% |
| Trimethoprim-sulfamethoxazole | 16 | 16 | 100% | 67 | 74 | 91% | 16 | 16 | 100% | 2 | 2 | 100% | 11 | 11 | 100% | 137 | 156 | 88% |
| Ampicillin | 16 | 16 | 100% | 63 | 70 | 90% | 15 | 16 | 94% | 2 | 2 | 100% | 11 | 11 | 100% | 135 | 155 | 87% |
| Chloramphenicol | 16 | 16 | 100% | 57 | 74 | 77% | 14 | 16 | 88% | 2 | 2 | 100% | 10 | 11 | 91% | 123 | 156 | 79% |
| Ciprofloxacin | 1 | 16 | 6% | 0 | 73 | 0% | 3 | 16 | 19% | 0 | 2 | 0% | 1 | 11 | 9% | 0 | 155 | 0% |
| Nalidixic acid | 1 | 16 | 6% | 6 | 73 | 8% | 2 | 16 | 13% | 0 | 2 | 0% | 3 | 11 | 27% | 26 | 155 | 17% |
| Tetracycline | 13 | 16 | 81% | 12 | 24 | 50% | 15 | 16 | 94% | 0 | 2 | 0% | 10 | 11 | 91% | 60 | 156 | 38% |
