# Supplementary material for: Exploration of risk factors for ceftriaxone resistance in invasive non-typhoidal Salmonella infections in western Kenya
Source: PLoS One. 2020 Mar 3;15(3):e0229581. doi: 10.1371/journal.pone.0229581 (PMC7053705; doi:10.1371/journal.pone.0229581)
Supplement: S1 Table — (DOCX) [file pone.0229581.s005.docx]

**S1 Table 1. Results of antimicrobial use survey conducted among health care-providers in Siaya county, Kenya, 2014.**

| **Responses** | **N=7** | **Percent**  **(%)** |
| --- | --- | --- |
| Third generation cephalosporin was prescribed at their hospital ward | 7 | (100) |
| Ceftriaxone was the most often prescribed 3rd generation cephalosporin | 7 | (100) |
| Prescription trend of 3rd generation cephalosporins since they became first available | | |
| Increasing | 4 | (57) |
| Did not change | 2 | (29) |
| Do not know | 1 | (14) |
| How often 3^rd^ generation cephalosporins were prescribed for children admitted with meningitis during the past year | | |
| Always | 5 | (71) |
| Often | 1 | (14) |
| Sometimes | 1 | (14) |
| How often 3^rd^ generation cephalosporins were prescribed for children admitted with sepsis during the past years | | |
| Always | 3 | (43) |
| Often | 4 | (57) |
| How often 3^rd^ generation cephalosporins were prescribed for children admitted with acute febrile illness during the past years | | |
| Often | 2 | (29) |
| Sometimes | 4 | (57) |
| Rarely | 1 | (14) |
| Third generation cephalosporin that was available at their ward on the day of interview^#^ | | |
| Ceftriaxone | 6 | (100) |
| How often commonly prescribed 3^rd^ generation cephalosporins were not been available during the past year | | |
| Often | 2 | (29) |
| Sometimes | 1 | (14) |
| Never | 4 | (57) |
| How often do the families of admitted patients have situations when they cannot buy a full dose of the prescribed 3rd generation cephalosporins? | | |
| Never | 2 | (29) |
| Often | 2 | (29) |
| Rarely | 2 | (29) |
| Sometimes | 1 | (14) |
| First and 2^nd^ generation cephalosporins were prescribed at their hospital ward | 5 | (71) |
| Other beta-lactam antimicrobials were prescribed at their ward | 7 | (100) |
| Other beta-lactam antimicrobials were available on the day of interview | 7 | (100) |
| Carbapenems been available at the hospital in the past year | 1 | (14) |
| Carbapenems were available on the day of interview | 0 | (0) |

^#^Missing variables were excluded from calculations
