# Supplementary material for: Exploration of risk factors for ceftriaxone resistance in invasive non-typhoidal Salmonella infections in western Kenya
Source: PLoS One. 2020 Mar 3;15(3):e0229581. doi: 10.1371/journal.pone.0229581 (PMC7053705; doi:10.1371/journal.pone.0229581)
Supplement: S2 Table — (DOCX) [file pone.0229581.s006.docx]

**S2 Table. Results of antimicrobial use survey conducted among pharmacies in Siaya county, Kenya, 2014**

| **Responses** | **N=23** | **Percent**  **(%)** |
| --- | --- | --- |
| Pharmacies that dispensed antimicrobials | 23 | (100%) |
| Pharmacies that sold 3^rd^ generation cephalosporins | 20 | (87) |
| Had ceftriaxone in stock on the day of interview | 16 | (80) |
| Had cefixime in stock on the day of interview | 6 | (30) |
| Had ceftazidime in stock on the day of interview | 2 | (10) |
| How often 3^rd^ generation cephalosporins were unavailable at their pharmacy during the last year | | |
| Often | 4 | (20) |
| Sometimes | 6 | (30) |
| Rarely | 5 | (25) |
| Never | 5 | (25) |
| Pharmacies that stocked other cephalosporins in the past year | 8 | (35) |
| How often other cephalosporins were unavailable at their pharmacy during the last year | | |
| Often | 2 | (25) |
| Sometimes | 1 | (13) |
| Rarely | 2 | (25) |
| Never | 3 | (38) |
| Pharmacies that stocked other beta-lactam antimicrobials in the past year | 23 | (100.0) |
| Pharmacies that had other beta-lactam antimicrobials available on day of interview | 22 | (96) |
| How often other beta-lactam antimicrobials were unavailable at their pharmacy during the last year | | |
| Sometimes | 4 | (17) |
| Rarely | 4 | (17) |
| Never | 15 | (65) |
| Pharmacies stocked ciprofloxacin in the past year | 21 | (91) |
| Pharmacies that had ciprofloxacin available on the day of interview | 19 | (91) |
| How often ciprofloxacin was unavailable at their pharmacy during the last year | | |
| Sometimes | 3 | (14) |
| Rarely | 2 | (10) |
| Never | 16 | (76) |
| Pharmacies that stocked cotrimoxazole in the past year | 23 | (100) |
| Pharmacies that had cotrimoxazole available on the day of interview | 21 | (91) |
| How often cotrimoxazole was unavailable in the past year | | |
| Sometimes | 3 | (13) |
| Rarely | 4 | (17) |
| Never | 16 | (70) |
| Pharmacies stocked gentamicin in the past year | 22 | (96) |
| Pharmacies that had gentamicin on day of interview | 19 | (86) |
| How often gentamicin was unavailable in the past year | | |
| Sometimes | 4 | (18) |
| Rarely | 4 | (18) |
| Never | 14 | (64) |
| Pharmacies that stocked Fansidar in the past year | 16 | (70) |
| Pharmacies that had Fansidar on the day of interview | 11 | (69) |
| How often Fansidar was unavailable in the past year |  |  |
| Often | 2 | (13) |
| Sometimes | 4 | (25) |
| Rarely | 5 | (31) |
| Never | 5 | (31) |
| Pharmacies that stocked carbapenems in the past year | 4 | (17) |
| Pharmacies that had carbapenems on the day of interview | 2 | (50) |
| Pharmacies that reported patients asking to purchase carbapenems | 4 | (17) |
| How often a patient cannot afford a full prescribed course of antimicrobials | | |
| Always | 5 | (22) |
| Often | 3 | (13) |
| Sometimes | 8 | (35) |
| Rarely | 6 | (26) |
| Never | 1 | (4) |
| Pharmacies that reported that they would sell a partial prescription to a customer | 13 | (57) |
| How often a customer would return to buy the remainder of the prescription | | |
| Always | 5 | (39) |
| Often | 5 | (39) |
| Sometimes | 3 | (23) |
