# Supplementary material for: Exploration of risk factors for ceftriaxone resistance in invasive non-typhoidal Salmonella infections in western Kenya
Source: PLoS One. 2020 Mar 3;15(3):e0229581. doi: 10.1371/journal.pone.0229581 (PMC7053705; doi:10.1371/journal.pone.0229581)
Supplement: S3 Table — (DOCX) [file pone.0229581.s007.docx]

**S3 Table. Results of antimicrobial use survey conducted among agrovets or shops that supply drugs, feeds, and other products for livestock, Siaya county, Kenya, 2014.**

| **Responses** | **N=15** | **Percent**  **(%)** |
| --- | --- | --- |
|  |  |  |
| Agrovets that sell animal health products | 15 | (100) |
| Agrovets that sell antimicrobials for use in animals | 15 | (100) |
| The main reason farmers purchase antimicrobials |  |  |
| for prophylaxis | 2 | (13) |
| for treatment | 13 | (87) |
| Agrovets that sell beta-lactam antimicrobials | 14 | (93) |
| for cattle | 14 | (100) |
| for sheep | 14 | (100) |
| for goats | 14 | (100) |
| for poultry | 1 | (7) |
| Agrovets that sell aminoglycosides | 5 | (33) |
| for cattle | 5 | (100) |
| for sheep | 5 | (100) |
| for goats | 5 | (100) |
| for poultry | 0 | (0.0) |
| Agrovets that sell tetracyclines | 13 | (87) |
| for cattle | 13 | (100) |
| for sheep | 13 | (100) |
| for goats | 13 | (100) |
| for poultry | 12 | (92) |
| Agrovets that sell quinolones | 2 | (13) |
| for cattle | 2 | (100) |
| for sheep | 2 | (100) |
| for goats | 2 | (100) |
| for poultry | 1 | (50) |
| Agrovets that sell macrolides | 13 | (87) |
| for cattle | 3 | (23) |
| for sheep | 3 | (23) |
| for goats | 4 | (31) |
| for poultry | 12 | (92) |
| Agrovets that sell sulphonamides | 15 | (100) |
| for cattle | 12 | (80) |
| for sheep | 12 | (80) |
| for goats | 12 | (80) |
| for poultry | 15 | (100) |
| Agrovets that sell cephalosporins | 3 | (20) |
| for cattle | 2 | (67) |
| Year when cephalosporin antimicrobials first become available in their shop | | |
| 2010 | 1 | (50) |
| 2013 | 1 | (50) |
| Agrovets that sell animal feeds | 14 | (93) |
| Sell poultry feeds | 14 | (100) |
| Sell cattle, sheep, and goat feeds | 11 | (79) |
| Agrovets that sell animal supplements | 14 | (93) |
| Sell poultry supplements | 14 | (100) |
| Sell cattle, sheep, and goat supplements | 12 | (86) |
