# Supplementary material for: Exploration of risk factors for ceftriaxone resistance in invasive non-typhoidal Salmonella infections in western Kenya
Source: PLoS One. 2020 Mar 3;15(3):e0229581. doi: 10.1371/journal.pone.0229581 (PMC7053705; doi:10.1371/journal.pone.0229581)
Supplement: S4 Table — (DOCX) [file pone.0229581.s008.docx]

| **Study** | **Number of isolates** | **Serotype** | **Antimicrobials to which the isolates were resistant** | **Resistance genes identified** |
| --- | --- | --- | --- | --- |
| CNS study | 2 | *Salmonella* Typhimurium | Ampicillin, ceftiofur, ceftriaxone, chloramphenicol, gentamycin, streptomycin, sulfisoxazole, tetracycline, and trimethoprim-sulfamethoxazole. | *bla*_CTX-M-15_ , *bla*_TEM-1_, *bla*_OXA-1_, *cat*A1, *cat*B3, *aac*(3)*-IIa*, *str*AB, *aad*A24, *sul1, sul2*, *tet*A, *dfrA1*, *dfrA14*, *aac(6')lb-cr* |
|  |  |  |  |  |
| Surveillance program | 2 | *Salmonella* Typhimurium | Ampicillin, ceftiofur, ceftriaxone, chloramphenicol, gentamycin, streptomycin, sulfisoxazole, tetracycline, trimethoprim-sulfamethoxazole, ciprofloxacin and nalidixic acid. | *bla*_CTX-M-15_ , *bla*_TEM-1_, *bla*_OXA-1_, *cat*A1, *cat*B3, *aac*(3)*-IIa*, *str*AB, *aad*A24, *sul1, sul2*, *tet*A, *dfrA1*, *dfrA14*, *aac(6')lb-cr*, *gyrA* |

**S4 Table. Serotyping and whole genome sequencing results of 4 selected non-typhoidal *Salmonella* isolates from invasive infections identified among central nervous system infection study (CNS study), and International Emerging Infections Program (surveillance program) participants during 2009–2014, Siaya county, Kenya**
